# Supplementary material for: A Subset of Non-Small Cell Lung Cancer Patients Treated with Pemetrexed Show 18F-Fluorothymidine “Flare” on Positron Emission Tomography
Source: Cancers (Basel). 2023 Jul 22;15(14):3718. doi: 10.3390/cancers15143718 (PMC10377924; doi:10.3390/cancers15143718)
Supplement: Supplementary file 1 [file cancers-15-03718-s001.zip › cancers-2392607-supplementary.pdf]

Supplementary figures
Figure S1

A

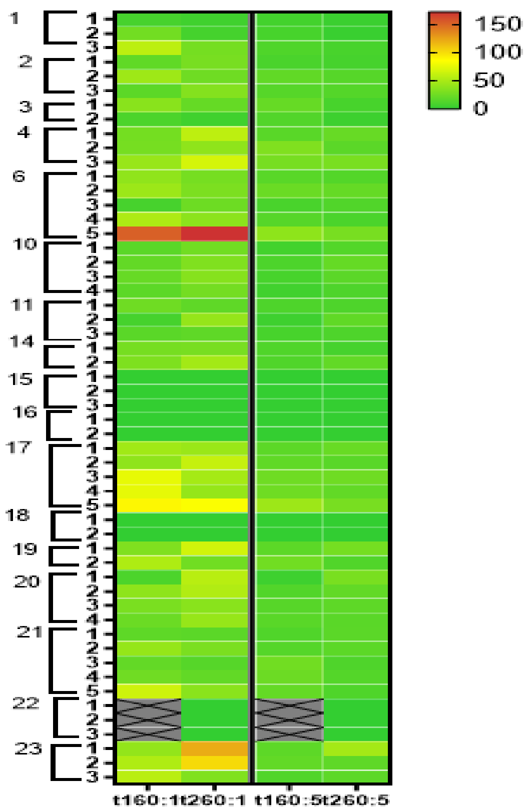

B

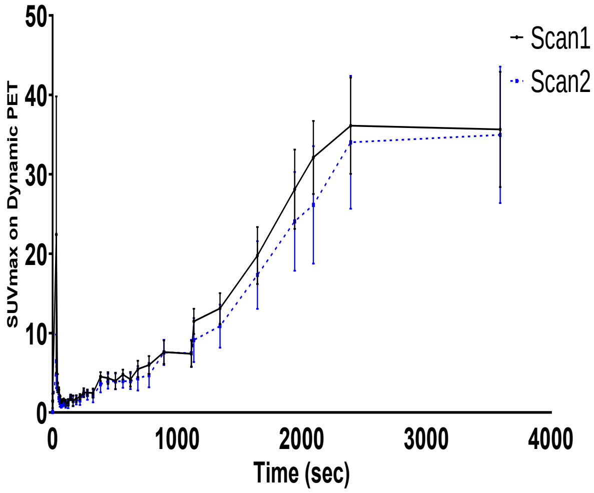

Figure S2

A

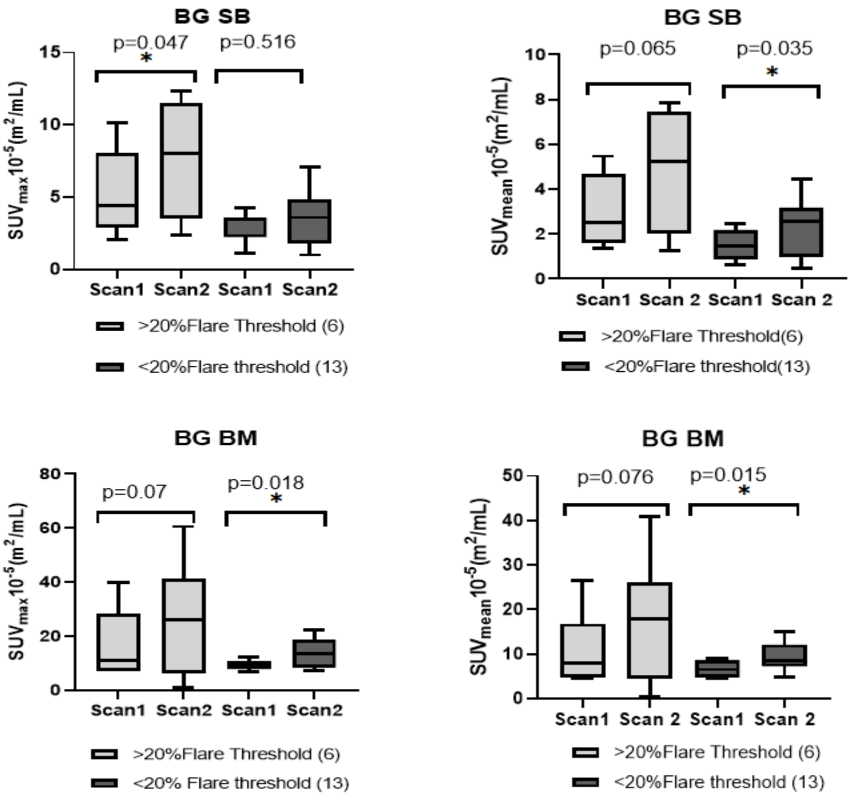
BG SB
p=0.047
p=0.516
SUVmax10-5(m2/mL)
Scan1 Scan2 Scan1 Scan2
>20%Flare Threshold (6)
<20%Flare threshold (13)
BG SB
p=0.065
p=0.035
SUVmean10-5(m2/mL)
Scan1 Scan 2 Scan1 Scan 2
>20%Flare Threshold(6)
<20%Flare threshold(13)
BG BM
p=0.07
p=0.018
SUVmax10-5(m2/mL)
Scan1 Scan2 Scan1 Scan2
>20%Flare Threshold (6)
<20% Flare threshold (13)
BG BM
p=0.076
p=0.015
SUVmean10-5(m2/mL)
Scan1 Scan 2 Scan1 Scan 2
>20%Flare Threshold (6)
<20%Flare threshold (13)

B

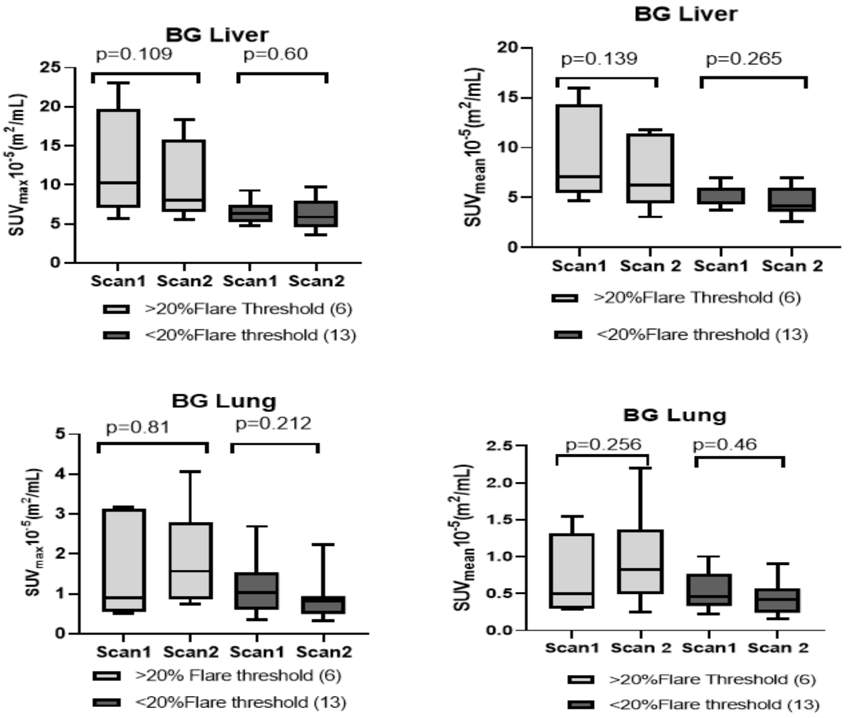
BG Liver
p=0.109
p=0.60
SUVmax10-5(m2/mL)
Scan1 Scan2 Scan1 Scan2
>20%Flare Threshold (6)
<20%Flare threshold (13)
BG Liver
p=0.139
p=0.265
SUVmean10-5(m2/mL)
Scan1 Scan 2 Scan1 Scan 2
>20%Flare Threshold (6)
<20%Flare threshold (13)
BG Lung
p=0.81
p=0.212
SUVmax10-5(m2/mL)
Scan1 Scan2 Scan1 Scan2
>20% Flare threshold (6)
<20%Flare threshold (13)
BG Lung
p=0.256
p=0.46
SUVmean10-5(m2/mL)
Scan1 Scan 2 Scan1 Scan 2
>20%Flare Threshold (6)
<20%Flare threshold (13)

Figure S1 ^{18}F -FLT PET uptake in the Dynamic images with outcome

A) Heatmaps showing the change in the SUV_{max} at baseline and 4h post pemetrexed on dynamic imaging for the first and 5th min into the scan. One patient had no evaluable dynamic PET images at baseline which is shown in grey. B) Graph of the TAC curves for SUV_{max} at baseline and 4h post pemetrexed on the dynamic ^{18}F-FLT-uptake scans

Figure S2 The variation in ^{18}F-FLT flare in TS responsive and non-TS responsive background tissues

A) Boxplot of the change in SUV_{max} and SUV_{mean} in the TS responsive heathy tissue: background small bowel and bone marrow for the group with ^{18}F-FLT-flare and no flare between the two-scan time points B) Boxplot of the change in SUV_{max} and SUV_{mean} in the non-TS responsive heathy tissue: background liver and lung for the group with ^{18}F-FLT flare and no flare between the two-scan time points. * represent significant p value.

Supplementary Table S1: Survival data of the study variables.

**Variables**	**Overall Survival(OS)**	**HR (95% CI)**	**Log-Rank P**
ΔSUV_{max}	(TTP)14.0m vs 6.0m	1.30 (1.20- 26.80)	0.709
ΔSUV_{max}	15.0m vs 31.0 m	0.48 (10.89-25.11)	0.152
Baseline Plasma TK1	15.0m vs 67.0m	5.19 (7.61-28.39)	0.020
∆ Plasma TK1	15.0m vs 15.0m	1.39 (9.12-20.88)	0.587
Baseline Plasma dUrd	29.0m vs 15.0 m	2.04(12.10-21.80)	0.208
∆ Plasma dUrd	20.0m vs 15.0 m	1.97(10.10-19.90)	0.361

Abbreviation: TTP: Time to progression, CI: Confidence Interval
